# Role of Intraoperative Documentation: Avoidable Ulnar Nerve Injury During Implant Removal

**DOI:** 10.7759/cureus.65701

**Published:** 2024-07-29

**Authors:** Amit Kale, Ayush Taneja, Ketan Kulkarni, Pankaj Sharma, Meet B Shah

**Affiliations:** 1 Orthopaedics, Dr. D. Y. Patil Medical College, Hospital and Research Centre, Dr. D. Y. Patil Vidyapeeth, Pune, IND

**Keywords:** operation notes, peripheral nerve repair, ortho surgery, implant removal, distal humerus fracture, ulnar nerve neuropathy

## Abstract

Injuries to the ulnar nerve during open reduction and internal fixation of distal humerus fractures are a well-known phenomenon. However, ulnar nerve injury during implant removal has not been well documented. We performed implant removal in a united distal humerus fracture with the aim of improving the elbow's range of motion. Even with proper surgical precautions in place, the ulnar nerve was damaged during dissection. This report aims to provide insight into this rare phenomenon, and the reasons for this injury are examined retrospectively. The importance of operation notes, the surgical approach, anterior transposition of the nerve, and how this and other factors could have helped the surgeons avoid this complication have also been highlighted.

## Introduction

Ulnar neuropathy post-surgical fixation of distal humerus fractures has an incidence that varies between 0% and 51%; on average, it is approximately 12% [1]. However, the majority of these studies are retrospective, and the presence of ulnar nerve dysfunction before the trauma is unknown [2]. The typical indications for implant removal include infection, implant exposure, implant migration, implants causing discomfort, to allow for normal bone growth in children, suspected future complications, to perform surgical release for stiffness, or at the request of the patient [3,4]. The true incidence of ulnar nerve injury in the case of revision surgery or implant removal is unknown. Implant removal requires the same surgical dissection as in the case of open reduction and internal fixation of the fracture. In some cases, this may be further complicated by the presence of adhesions, fibrosis, heterotopic ossification, and the presence of implants from the previous surgery [4]. Hence, the authors believe that the incidence of such surgical complications must be higher than reported.

Cases of ulnar nerve injury in a secondary procedure, such as revision internal fixation for nonunion, have been reported [5]. However, damage to the ulnar nerve in a sole implant removal procedure following fracture union has not been documented.

This study aims to bring awareness to surgeons regarding this rarely seen intraoperative complication during implant removal, emphasize once more the importance that operation notes from the previous surgery hold, and retrospectively examine the steps that could have been taken to prevent this injury.

## Case presentation

**Figure 1:**
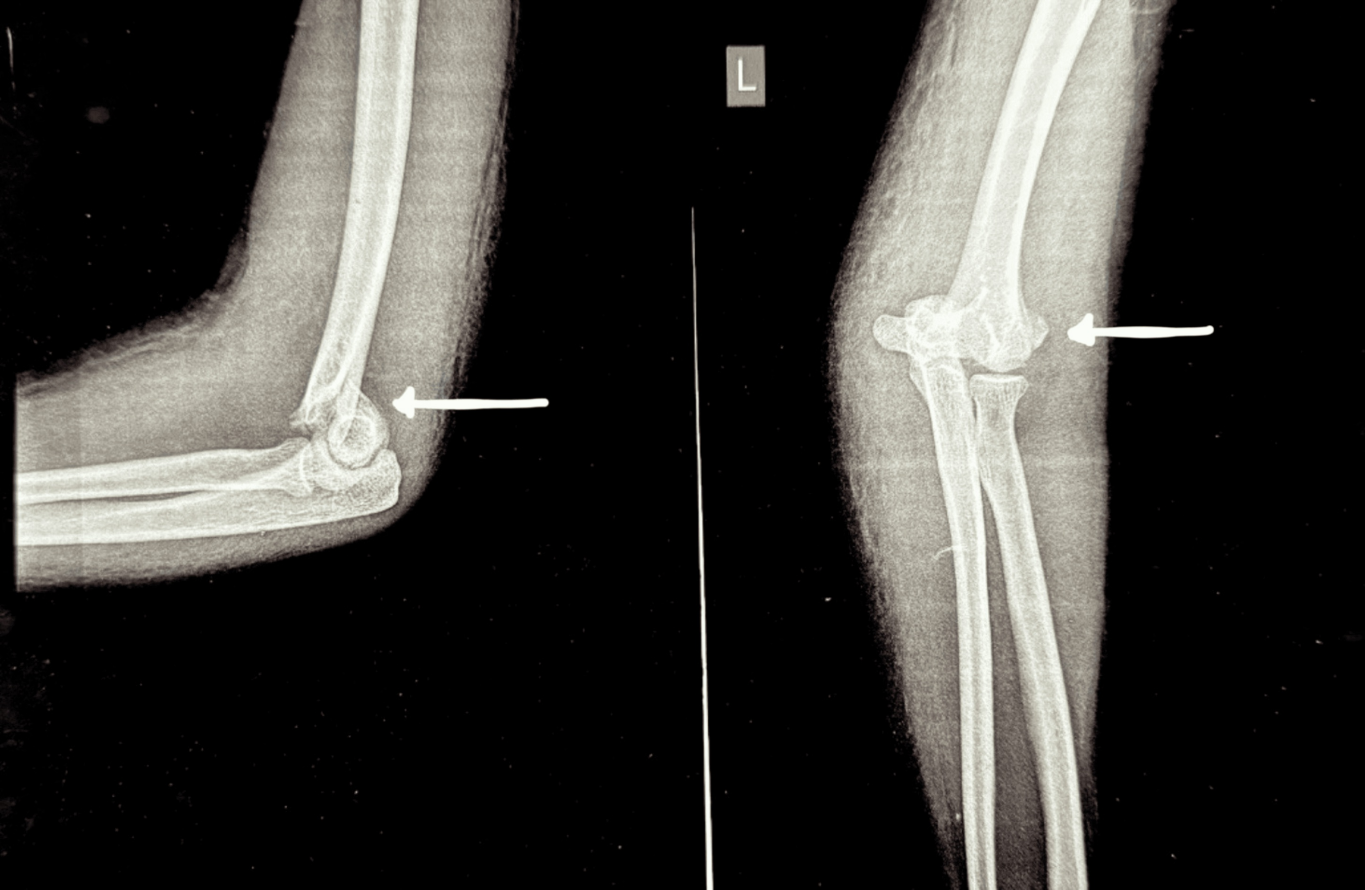
Anteroposterior and lateral radiographs of the left elbow showing a distal humerus fracture

**Figure 2:**
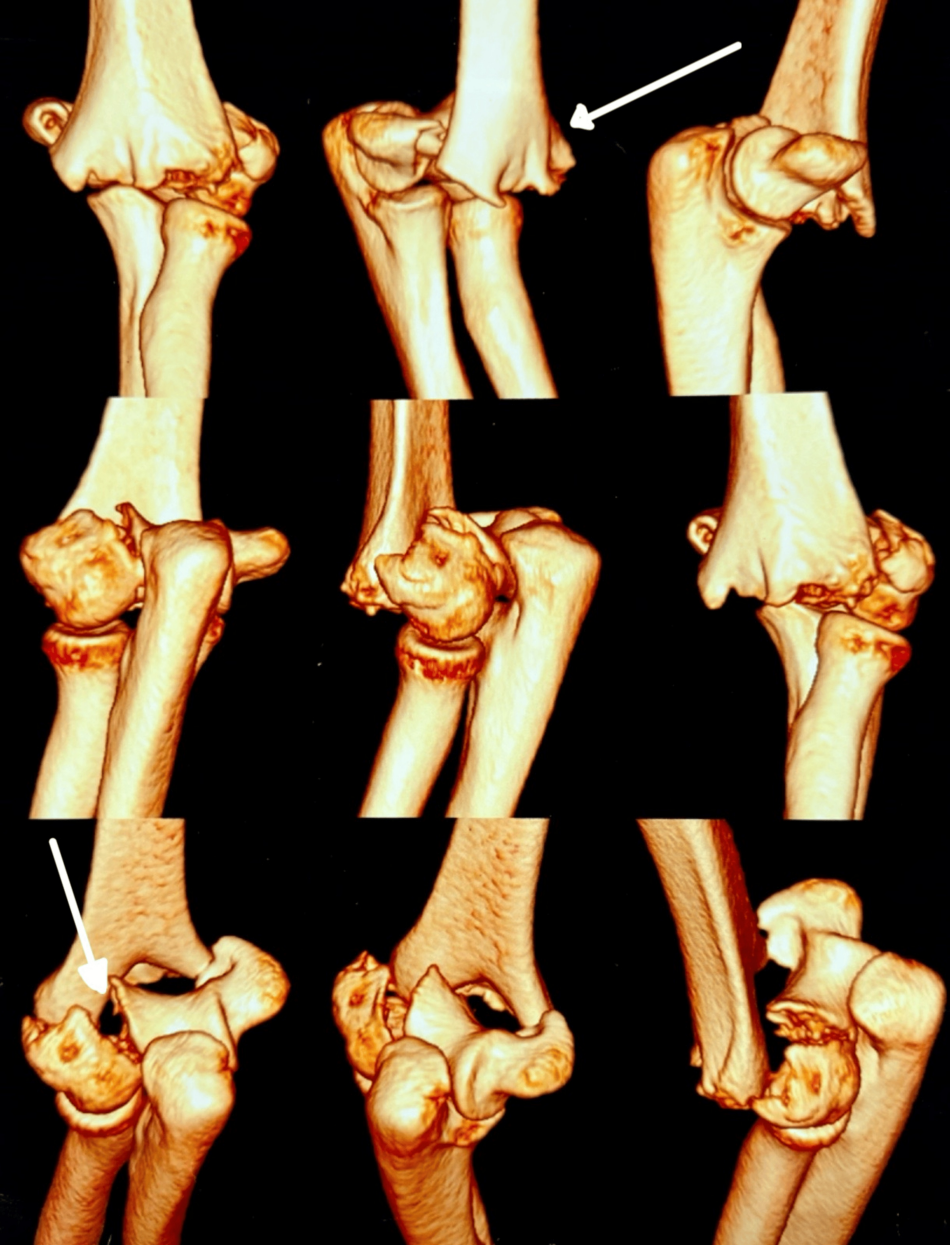
Three-dimensional computed tomography of the left elbow showing a distal humerus intra-articular fracture

An informed consent (type 2) was obtained from the patient's guardian for the publication of this case report. A 13-year-old female patient presented to the emergency department complaining of pain, diffuse swelling, and restricted movements of the left elbow. The patient did not have any neurovascular compromise on presentation. The patient had been operated on in an external, rural setting for a distal humerus fracture eight months ago. The surgical scar suggested a posterior approach had been used, which had been extended distally over the medial aspect of the olecranon. The patient had an elbow flexion-extension range of 30-120° and a full range of motion (ROM) for pronation and supination. The preoperative radiograph and CT showed a distal humerus, type C1 (AO/OTA classification) fracture, which is characterized as a complete articular simple metaphyseal fracture (Figures 1, 2).

**Figure 3:**
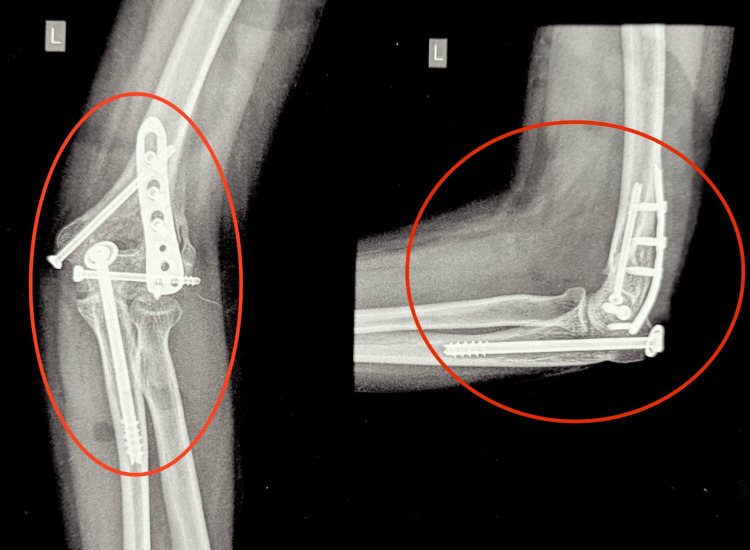
Anteroposterior and lateral radiographs of the left elbow on presentation after primary surgery with implants in situ

The patient did not have any other documents detailing her previous surgery. A new radiograph of her left elbow was performed, which showed a united distal humerus fracture with distal humerus dorsolateral plate, medial column, and transcondylar CC screws in situ and a CC screw, which had been used to fix the olecranon osteotomy (Figure 3).

**Figure 4:**
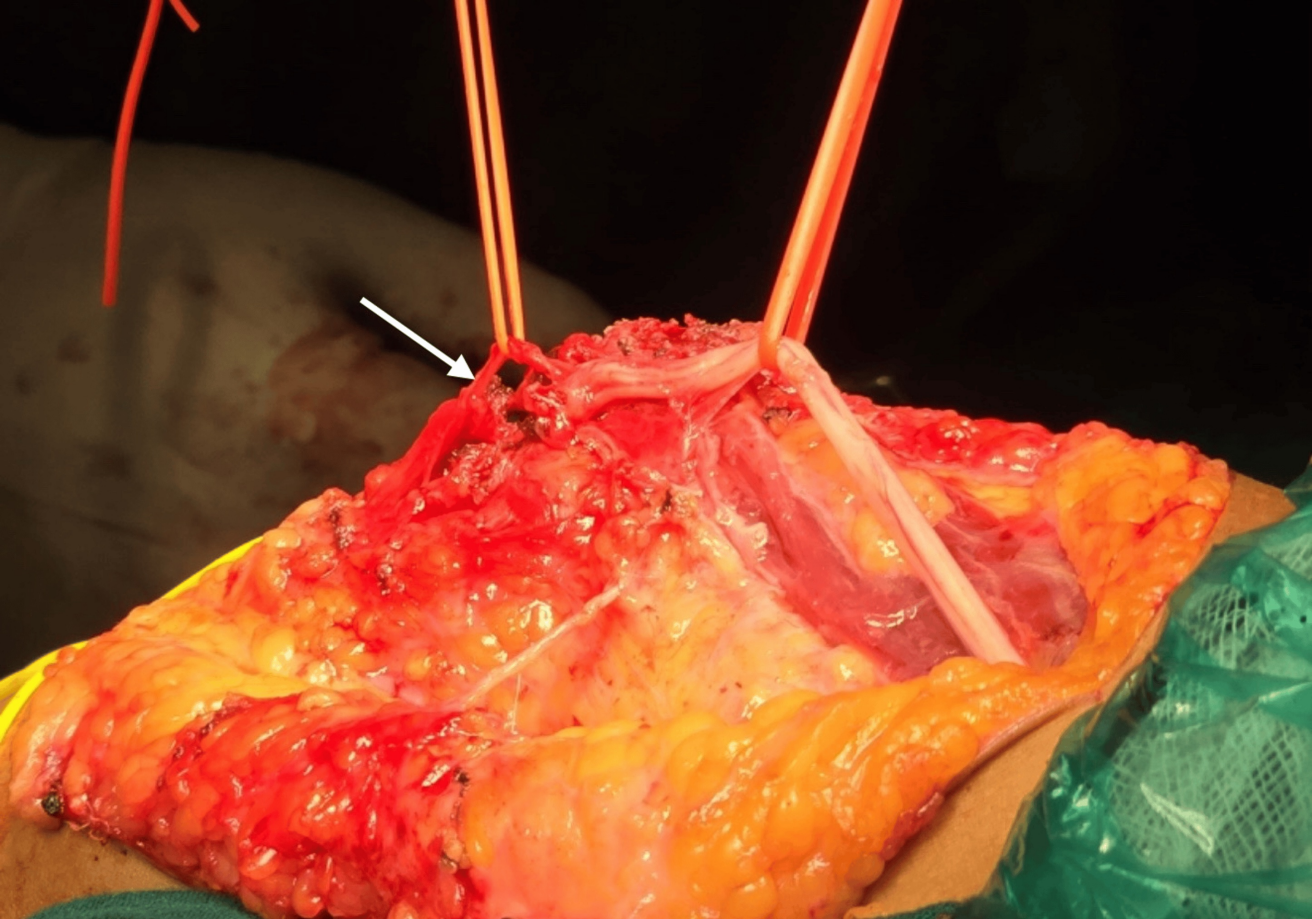
Intraoperative image showing the incompletely transected ulnar nerve

The team opted for complete hardware removal to improve elbow extension. The decision was based on the patient's limited ROM and the successful union of the fracture. The patient was taken up for implant removal under general anesthesia. The patient was placed in a lateral position with a sterile tourniquet, and the incision was taken over the previous surgical scar. A superficial dissection was performed, and a callus with fibrosis was noted at the fracture site. The olecranon CC screw with the washer was identified and removed. The lateral column plate was exposed and removed. The callus over the medial aspect was removed to expose the medial column CC screw, which was subsequently removed. The ulnar nerve could not be identified in its anatomical location. The transcondylar CC screw was identified. However, while exposing this CC screw, the ulnar nerve was damaged by the dissection scissors (Figure 4).

**Figure 5:**
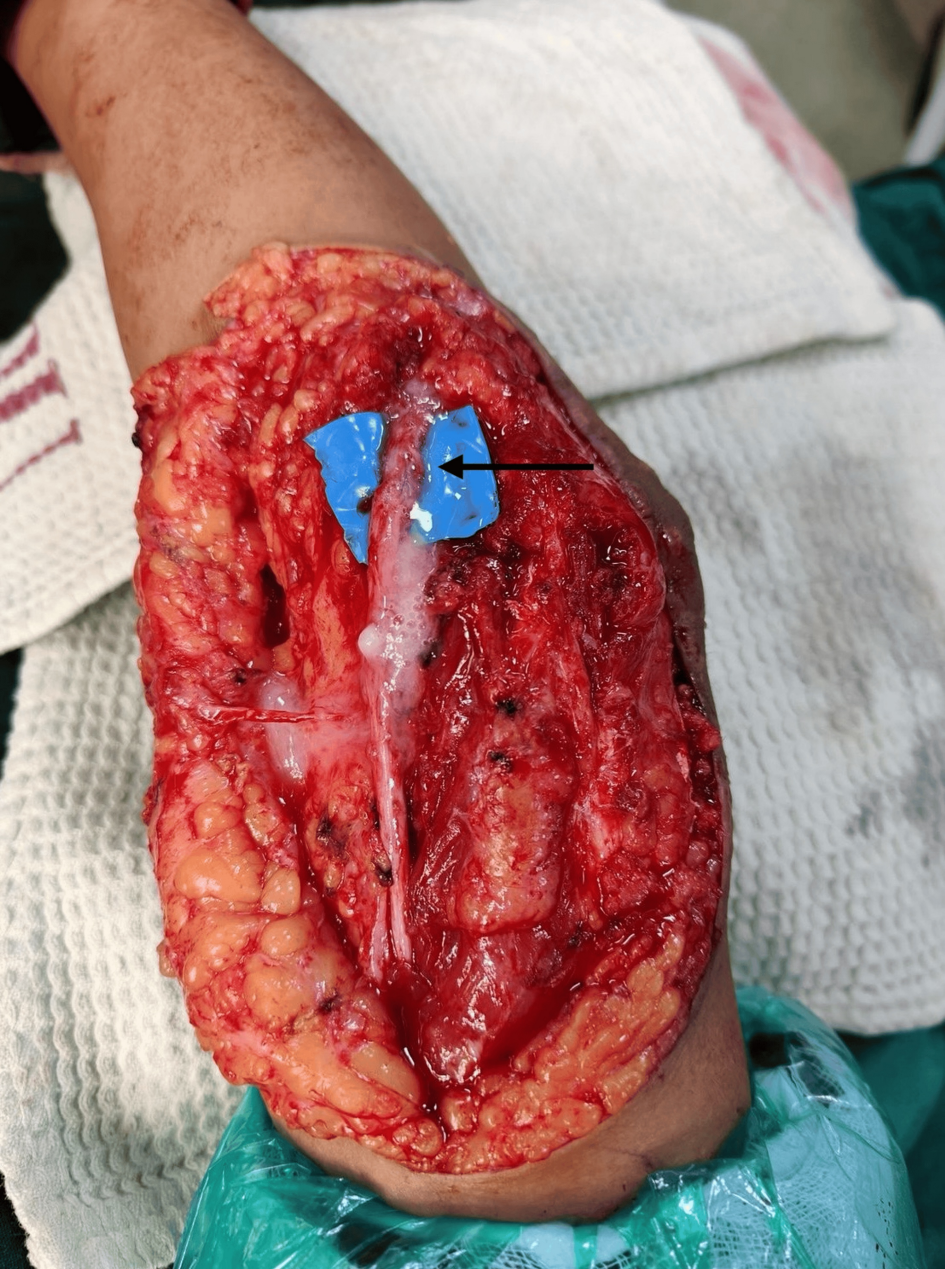
Intraoperative image after the surgical repair of the ulnar nerve using Prolene sutures (Ethicon, Bridgewater, New Jersey) and fibrin glue

The nerve was partially transected. Fibrosis and adhesions of the nerve to the surrounding structures were noted. The screw was then removed, and the elbow ROM was checked. Plastic surgery was then involved in ulnar nerve repair. The nerve was explored proximally until the medial intermuscular septum and the distal end were explored and mobilized until the distal branches to the flexor carpi ulnaris. The cut ends of the nerve fascicles were freshened, and the fascicular repair was done using synthetic polypropylene 7-0 sutures and fibrin glue with the elbow in extension (Figure 5).

**Figure 6:**
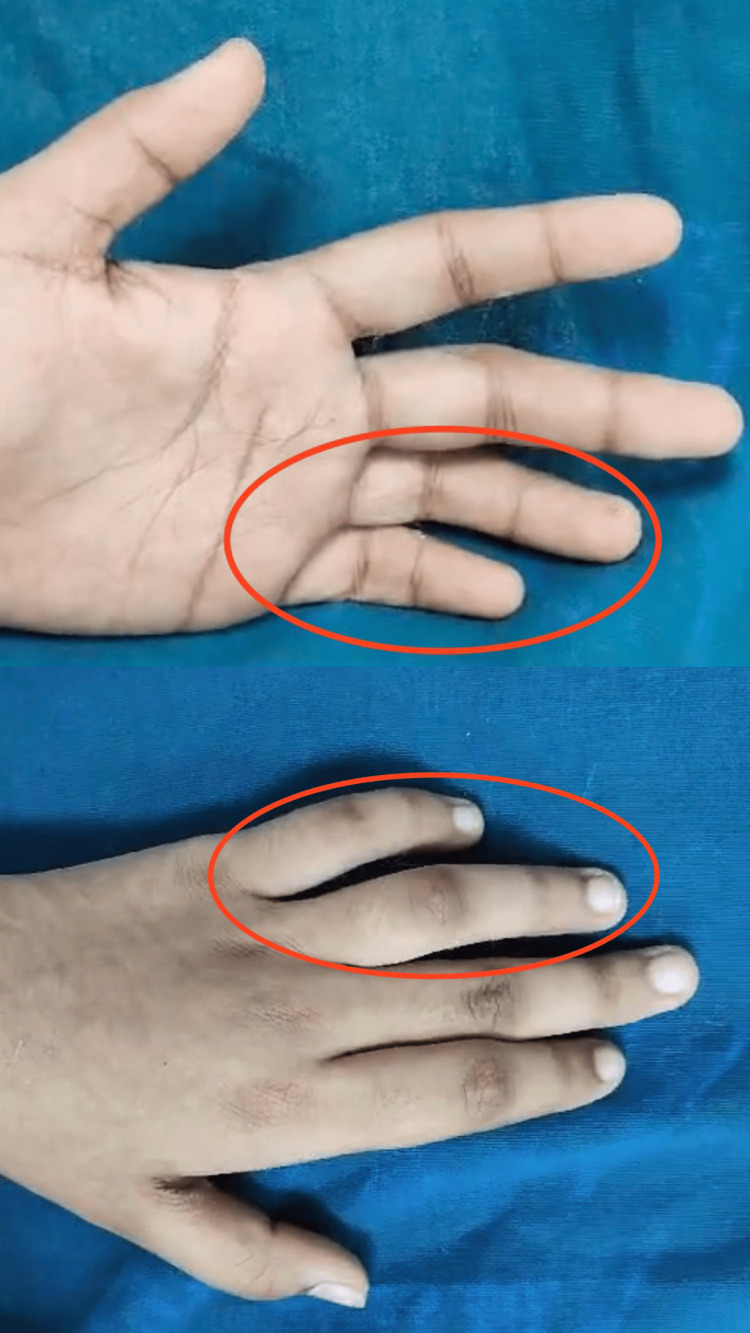
Postoperative clawing of the ulnar two fingers

The nerve was then transposed anteriorly. A thorough wash was given with normal saline, and a surgical suction drain was placed. Closure was performed in layers, and the limb was immobilized with an above-elbow slab with the wrist and hand in the functional position. Immediately postoperatively, the patient had mild clawing and reduced sensations over the ulnar two digits (Figure 6).

The patient now displays loss of intrinsic muscle function with loss of abduction, adduction, and opposition of the same digits. The limb will be immobilized with the wrist in 30° of extension, 45° of flexion at the metacarpophalangeal (MCP) joint, and 15° of flexion at the proximal interphalangeal (PIP) and distal interphalangeal (DIP) joints for another two months. The plastic surgeons will plan further management based on the recovery achieved at the three- and six-month follow-up.

## Discussion

Ulnar nerve injuries during primary procedures for elbow trauma are well documented. However, the incidence of injuries during hardware removal remains unknown. We suspect this compilation is underreported as removal requires surgical dissection, which is even more difficult due to the scar tissue presence from the primary operation.

Distal humerus fractures, which require open reduction and internal fixation, often involve mobilizing the ulnar nerve for fracture reduction and fixation with plates and screws [6-8]. This maneuver involves a risk of damage to the ulnar nerve. Many cases of ulnar nerve injury may involve transient paresthesia or numbness, but motor function might not be affected [9-11]. Some cases may involve severe neuropathy with loss of hand function and substantial muscle weakness, which can greatly compromise the final patient outcome after a distal humerus fracture [12].

Donders et al., in a series of 62 patients with nonunion of the distal humerus treated with internal fixation, showed that new ulnar neuropathies were present in five patients postoperatively. However, it is unclear whether the patients with new ulnar neuropathies had undergone previous internal fixation, which implants were used, and whether the ulnar nerve had been anteriorly transposed during the previous procedure [13].

Considering that many surgeons advocate for routinely performing an anterior transposition of the ulnar nerve during surgical fixation of distal humerus fractures, this shows the concern that surgeons have relating to this complication, even though the incidence of such nerve palsy postoperatively is not explicitly described in the literature [14-17]. In theory, the advantages of transposition include preventing kinking, moving the nerve away from the implants, avoiding subluxation of the nerve over the medial epicondyle, limiting the entrapment potential in the scar, and allowing for better excursion of the nerve. Contrary to this, some surgeons believe that the risk of ulnar nerve dysfunction increases with this extensive nerve manipulation and handling [16].

Wang et al. postulated through their small cohort of 20 patients that anterior transposition of the ulnar nerve prevented ulnar neuropathy and was necessary. In the 20 cases, they described a standard posterior approach with an olecranon osteotomy being used and the ulnar nerve being anteriorly transposed subcutaneously. They found no symptoms of ulnar neuropathy [14].

A detailed operation note from the previous surgery could have played a major role in preventing this injury in our case. Had the surgeons had prior knowledge of the precise location of the ulnar nerve in relation to the surrounding anatomical structures, additional attention could have been paid to the careful dissection of the nerve in the expected location. A detailed, accurate, and legible operation note is highly important for every surgeon. This is required for clear data transfer from the perioperative period to surgeons who might encounter the patient in the postoperative period. This contributes greatly to providing better safety and care to the patient. Its benefits as a tool for medicolegal, research, and audit purposes cannot be understated. Despite widespread recognition of its significance, up to 45% of operation documentation is inadequate and indefensible medicolegally [18].

In hindsight, we postulate that an anterior transposition of the ulnar nerve in the primary surgery could have reduced the chances of injury during the implant removal procedure. The nerve would not have been in close proximity to the implants, and it may have also helped to avoid adhesions of the nerve to the surrounding structures. The previous surgical incision had been extended over the medial aspect of the olecranon as opposed to the universal posterior approach to the elbow, where the incision is extended over the lateral aspect of the tip of the olecranon [19]. The authors suspect that this decision may have contributed to the formation of fibrosis around the ulnar nerve, which led to the difficult dissection during the implant removal procedure. The presence of readily available plastic and reconstructive surgeons in our tertiary care center was immensely valuable. Thus, we were able to minimize the damage and make an immediate attempt at nerve repair. There is a requirement for prospective studies on the occurrence of ulnar nerve injuries during hardware removal, with focused pre- and postoperative examinations, which include electromyographic evaluation and nerve conduction studies, as this would help in the accurate identification of cases. Details regarding whether the surgeons had proper data regarding the previous surgery should also be collected. This may also help us gain a better insight into the reasons for iatrogenic ulnar nerve injuries and how to prevent them.

## Conclusions

Ulnar nerve injury during an implant removal procedure for the distal humerus is a complication that we believe is underreported. The absence of operation notes from the previous surgery, the choice of the approach used, the surgeon's expertise, and the decision not to perform an anterior transposition of the ulnar nerve during the primary procedure may have played a role in the outcome. Early detection of damage to the ulnar nerve and the presence of plastic and reconstructive surgeons also play a crucial role in minimizing the damage and performing an adequate repair. The authors also suggest the use of an intraoperative nerve stimulator device to improve outcomes in cases where the localization of nerves during dissection is difficult. Future studies on the subject are warranted, specifically long-term prospective studies, to have a better understanding of the etiology and management of this complication.
